# Phytochemistry Profile, Antimicrobial and Antitumor Potential of the Methanolic Extract of *Tabernaemontana catharinensis* A DC and *Eragrostis plana* NEES

**DOI:** 10.1155/2024/5513141

**Published:** 2024-01-03

**Authors:** Emanoeli da Rosa, Cheila Denise Ottonelli Stopiglia, Michel Mansur Machado, Augusto Cezar Dotta Filho, Ursula Paula Reno Soci, Andreas Sebastian Loureiro Mendez, Tiago Fernandes, Edilamar Menezes de Oliveira, Cleci Menezes Moreira

**Affiliations:** ^1^Programa de Pós-Graduação em Bioquímica, Universidade Federal do Pampa-UNIPAMPA, Uruguaiana, RS, Brazil; ^2^Programa de Pós-Graduação em Ciências Farmacêuticas, Universidade Federal do Pampa-UNIPAMPA, Uruguaiana, RS, Brazil; ^3^Laboratório de Bioquímica e Biologia Molecular do Exercício, Escola de Educação Física e Esporte, Universidade de São Paulo-USP, São Paulo, SP, Brazil; ^4^Programa de Pós-Graduação em Ciências Farmacêuticas, Universidade Federal do Rio Grande do Sul-UFRGS, Porto Alegre, RS, Brazil

## Abstract

Natural compounds that have the potential to act as antimicrobials and antitumors are a constant search in the field of pharmacotherapy. *Eragrostis plana* NEES (Poaceae) is a grass with high allelopathic potential. Allelopathy is associated with compounds generated in the primary and secondary metabolism of the plant, which act to protect it from phytopathogens. *Tabernaemontana catharinensis* A DC (Apocynaceae), a tree in which its leaves and bark are used for the preparation of extracts and infusions that have anti-inflammatory and antinociceptive effects, is attributed to its phytochemical constitution. The objective of this study was to elucidate the phytochemical constitution, the antibacterial potential, the toxicity against immune system cells, hemolytic potential, and antitumor effect of methanolic extracts of *E. plana* and *T. catharinensis.* The phytochemical investigation was carried out using the UHPLC-QTOF MS equipment. The antibacterial activity was tested using the broth microdilution plate assay, against Gram-negative and Gram-positive strains, and cytotoxicity assays were performed on human peripheral blood mononuclear cells (PBMC) and *in vitro* hemolysis. Antitumor activity was performed against the colon cancer cell line (CT26). Results were expressed as mean and standard deviation and analyzed by ANOVA. *p* < 0.05 was considered significant. More than 19 possible phytochemical constituents were identified for each plant, with emphasis on phenolic compounds (acids: vanillic, caffeic, and quinic) and alkaloids (alstovenine, rhyncophylline, amezepine, voacangine, and coronaridine). Both extracts showed antibacterial activity at concentrations below 500 *µ*g/mL and were able to decrease the viability of CT26 at concentrations below 2000 *µ*g/mL, without showing cytotoxic effect on PBMCs and *in vitro* hemolysis at the highest concentration tested. This is the first report of the activity of *E. plana* and *T. catharinensis* extracts against colon cancer cell line (CT26). Studies should be carried out to verify possible molecular targets involved in the antitumor effect *in vivo*.

## 1. Introduction

The significant increase in drug-resistant pathogens is associated with the inappropriate and indiscriminate prescription of drugs used in clinical practice, treatment time, among other factors, and although there is a wide range of antimicrobial drugs on the market, resistance is a limiting factor, considered a major public health problem [1, 2].

Another problem that has a great impact on public health is the increasing number of cancer cases. It is estimated that more than 18 million cases are diagnosed each year [3]. Colon cancer, or colorectal cancer (CRC), appears among the three main types of cancer diagnosed in the population, affecting both sexes, in different life cycles; however, data from the literature support that the highest incidence occurs from 50 years old [3, 4].

Treatment for CRC has evolved tremendously in recent decades, with the removal of tumors being the first form of treatment, hence the importance of obtaining an early diagnosis. The surgical procedure can be curative, depending on the stage of the tumor [5]. When the removal procedure is not the best option, chemotherapy and/or radiotherapy treatment is necessary as adjuvants which, although they have evolved with the advancement of new drugs being developed, is still a high-cost treatment that culminates in the deterioration of physiological functions of the individual [6, 7]. There is a constant search for new therapeutic alternatives for both antimicrobial and anticancer agents, and herbal medicine is among their agents.

The use of plants as an alternative treatment for diseases is an ancient practice, and it is estimated that more than 80% of the world's population has already made use of some type of plant. With a healing purpose, Brazil has one of the greatest plant diversity in its flora in the world, and a large part is used for therapeutic purposes, many with scientific evidence of their effectiveness and a wide range still being studied [8]. The advance in the discovery of active compounds in products resulting from the secondary metabolism of plants brought a new perspective for the treatment of numerous pathologies, as well as clarification on the toxic potential of species used indiscriminately [9, 10].

*Eragrostis plana* NESS (Lovegrass) popularly known as “annoni” (Poaceae) is a grass originally from South Africa, brought to Brazil by accident in the 1950s, mixed with pasture seeds [11]. Initially seen as a promising pasture due to its great resistance to climate change, the annoni grass quickly spread through the fields of southern Brazil, due to its easy propagation. However, the predominance in soil compared to native species of the Gaucho pampa and the low nutritional value made it a problem for the agricultural sector [11], having its sale was banned in 1979. Since the prohibition of the sale of lovegrass seeds, studies involving growing this type of plant are mostly focused on the area of agrarian sciences, with the aim of trying to control its dissemination. Lovegrass has high resistance even to desiccant products, and its small seeds spread easily, making it practically impossible to control the pest around the soil.

The allelopathic profile of this dominant plant is due to its chemical constituents and secondary metabolites, which have different functions in the plant, such as nutrition and protection against insects [12]. Isolated fractions, containing diterpenes, obtained through the plant extract (*Eragrostis plana*) show action against phytofungi, justifying the studies aimed at the development of natural defensive agriculture taking advantage of the allelochemical potential of the plant [13].

*Tabernaemontana catharinensis* A DC, popularly known as “cobrina,” is a bushy plant, belonging to the Apocynaceae family, widely found in the southern region of Brazil, as well as in neighboring countries such as Uruguay, Argentina, and Paraguay [14].

The medicinal use of infusions and macerations of different parts of the plant has been reported for a long time, commonly used by indigenous civilizations as an antiophidic, and this property gives rise to its popular name (“snake = cobra in Portuguese”). The antinociceptive effect of the plant made it popularly used to relieve pain. Many activities attributed to *T. catharinensis* have already been scientifically proven. Some of its healing properties are anti-inflammatory, analgesic, antifungal, antioxidant, and even antineoplastic activity [15–18].

The therapeutic properties are related to its rich phytochemical constitution, mainly to the numerous indole alkaloids, which are reported in leaves, flowers, bark, and roots of the plant [19–22].

Fractions containing indole alkaloids isolated from *T. catharinensis* were tested against human laryngeal epithelial carcinoma cell line (Hep-2) [23] U251 (glioma), MCF-7 (breast), NCI-ADR/RES (drug-resistant ovary), 786-0 (kidney), NCI-H460 (lung), HT-29 (colon), K562 (leukemia), and PC-3 (prostate), showing a cytotoxic effect on the tested strains while not causing damage to viability and proliferation in a nontumor keratinocyte cell line (HaCat) [24]. Pereira et al. [25] obtained inhibition of cell growth when testing the extract fraction rich in alkaloids against breast, lung, prostate, and kidney cancer strains, emphasizing the plant`s anticancer potential.

The therapeutic effect of plant extracts is related to their phytochemical constituents, either raw or isolated. Both plants, *T. catharinensis* [16, 26–31] and *E. plana* [12, 32], have therapeutic effects already described and attributed to their phytochemical composition; however, there are few research using crude extract of *T. catharinensis*, and no reports for *E. plana* use against the CT26 tumor lineage, justifying the present study.

In this sense, the aim of this research was to evaluate the activity of crude methanolic extract of *Eragrostis plana* NEES and *Tabernaemontana catharinensis* A DC against different bacterial strains, test its cytotoxic effectiveness against colon cancer strain (CT26), and in contrast to assessing whether the plant exerts a toxic effect on cell culture of the peripheral blood mononuclear cell (PBMCs), and identify possible phytochemical constituents.

## 2. Materials and Methods

### 2.1. Chemicals

Methyl alcohol (CAS: 67-56-1, Merck®) for preparing the extracts and Histopaque-1077® and reagents for cell culture, including RPMI 1640 medium, fetal bovine serum (FBS), and penicillin/streptomycin, were purchased from Sigma-Aldrich (St. Louis, MO, USA). All other chemicals were of analytical grade and obtained from standard commercial suppliers.

### 2.2. Plants Materials

The plant material was collected in a municipality on the West frontier of the state of Rio Grande do Sul, lat. 29°08′45″S, long. 56°25′50″W (*E. plana),* and central region of the state, lat. 29°23′23″S, long. 53°24′24″W (*T. catharinensis)*, Brazil, with a sample being sent to the herbarium in São Gabriel-RS, cataloged with the numbers: HBEI 1660 to *Tabernaemontana catharinensis* A DC and HBEI 1661 to *Eragrostis plana* NEES.

### 2.3. Extract Preparation

Aerial parts of the plants (*E. plana)* and leaves (*T. catharinensis)* were used, previously washed, dried, and grounded, and then subjected to infusion (plant material: solvent; 1 : 10) in methyl alcohol, at a concentration of 70%, for the duration of five days. Afterward, the extract was filtered and kept in an oven at 37°C, until complete evaporation of the solvent, lyophilized, and kept under refrigeration for use.

### 2.4. Phytochemical Analysis

The phytochemical analysis was carried out at the Institute of Chemistry of the Federal University of Rio Grande do Sul (UFRGS). Analysis was performed using the UHPLC-QTOF MS, UHPLC system (Shimadzu-Nexera x2) equipped with the Shim-pack XR-ODS III column (2.0 × 50 mm, 1.6 *μ*m) Shimadzu®, the column was maintained at 35°C, and the QTOF-MS mass analyzer (Impact II, Bruker Daltonics). The QTOF-MS system is equipped with an electrospray ionization (ESI) source, operating in both positive and negative modes. The separation method was in gradient mode A: acetonitrile (0.1% formic acid) and B: aqueous phase (0.1% formic acid), the elution gradient started with 5% A maintained for 2 min, increasing to 95% up to 10 min, and maintained for 3 min, after which it was decreased to 5% in 2 min, and maintained for 5 min. The flow rate was 0.3 mL·min^−1^, and the injection volume was 10 *μ*L.

The operating parameters of the ESI were capillary voltage, 2500 V; end plate offset, 500 V; nebulizer pressure, 3 bar (N_2_); drying gas, 9 L·min^−1^ (N_2_); and drying temperature, 200°C, for negative mode, and capillary voltage, 4000 V; end plate offset, 500 V; nebulizer pressure, 3 bar (N_2_); drying gas, 9 L·min^−1^ (N_2_); drying temperature, 200°C, for positive mode. The QTOF-MS system operated in broadband collision-induced dissociation (bbCID) acquisition mode in a range of m/z 60−1600 with an acquisition rate of 2 Hz, and bbCID mode provides MS and MS/MS information at the same time. Calibration of the QTOF-MS system was performed with a sodium formate solution. The data were processed using Data Analysis 4.2 software. The mass accuracy tolerance was less than 5 ppm.

### 2.5. Antibacterial Activity Assay

To assess the minimal inhibitory concentration (MIC), the 96-well plate microdilution was used, containing Mueller Hinton broth [33]. Ten different concentrations of extracts were evaluated against different microorganisms to determine the lowest concentration capable of inhibiting bacterial growth.

Both extracts were tested against 13 microorganisms (MO): *Acinetobacter baumannii Bouvet and Grimont* ATCC® BAA-747; *Pseudomonas aeruginosa* (Schroeter) Migula ATCC® 27853; *Shigella* spp (clinical isolate); *Klebsiella pneumoniae* subsp. *pneumoniae* ATCC® 700603; *Proteus mirabilis* (clinical isolate); *Salmonella* spp (clinical isolate); *Staphylococcus aureus* subsp. ATCC® *aureus* 25923 and ATCC® bAA-750; *Enterococcus faecalis* ATCC® 29212; *Enterococcus casseliflavus* ATCC® 700327; *Escherichia coli* ATCC® 25922; *Streptococcus pyogenes* (clinical isolate).

The inocula were standardized in a spectrophotometer, with optical density between 0.08 and 0.13, reading in 600 nm, with approximately 1.10^8^ colony-forming units/mL, according to the 0.5 McFarland scale.

Extracts were serial diluted in decreasing concentration from 500 to 0.97 *μ*g/mL against 13 MO in a 96-well plate containing Mueller Hinton broth (Sigma-Aldrich®) in 100 *μ*L. Five microliters of inocula in Mueller Hinton broth were added. After 24 h of incubation in a bacteriological incubator (35°C), 20 *µ*L of 70% ethanol solution of 2,3,5-triphenyl-tetrazolium chloride (CTT) at 0.5% was added in each well. Then, the plates were incubated again for 120 minutes, at 35°C. The CTT has the ability to react with MO which has some metabolic activity, changing its color in the medium to red when the microorganism is viable, allowing visual differentiation.

### 2.6. *In Vitro* Hemolysis Assay

For this test, erythrocytes were obtained from 3 mL of human venous blood, collected in a heparinized Vacutainer® (approved by the Research Ethics Committee of the Universidade Federal do Pampa, nº 27045614.0.0000.5323), separated by centrifugation (1000 × *g* for 5 minutes), washed, centrifuged three times with saline solution, and then suspended in saline solution forming a hematocrit of 1%. The lyophilized extracts were resuspended in 0.9% saline solution, at a concentration of 2000 *μ*g/mL. This solution was filtrate; 2 mL of the filtrate was mixed with 1 mL of the erythrocyte suspension, with subsequent incubation under continuous homogenization for 20 minutes at room temperature [34]. Saline solution was used as negative control and distilled water as positive control. 

The occurrence of erythrocyte lysis was observed after centrifugation (1000 × *g* for 5 minutes). The results obtained were determined through reading in a spectrophotometer at 530 nm, being considered as capable or not of promoting hemolytic action *in vitro.* Tests were performed in triplicate. The average of the optical density (OD) readings was used in the following equation, by which it is possible to measure the % of *in vitro* hemolysis.

(1) $$\begin{array}{c} \text{Hemolysis}\left(\%\right):\frac{\left({\text{OD}}_{\text{test}}-{\text{OD}}_{\text{NC}}\right)}{\left({\text{OD}}_{\text{PC}}-{\text{OD}}_{\text{NC}}\right)}*100\%. \end{array}$$

### 2.7. Cell Culture and Viability

PBMC's culture was prepared by collecting 10 mL of human venous blood and collected in a heparinized Vacutainer® (approved by the Research Ethics Committee of the Universidade Federal do Pampa, no 27045614.0.0000.5323). PBMCs were isolated with Histopaque-1077® (Sigma-Aldrich®) and transferred to RPMI 1640 culture medium supplemented with 10% fetal bovine serum and 1% streptomycin/penicillin. The cells in suspension were kept in an incubator with a controlled environment of 37°C, 5% CO_2_, and then treated with the plant's extract at concentrations of 2000, 1000, 500, and 50 *μ*g/mL; hydrogen peroxide was used as a positive control (H_2_O_2_ 10 *µ*M). After 24 h of treatment, cell viability was assessed using the trypan blue exclusion technique [35].

The *in vitro* culture of colon cancer CT26-WT (ATCC® CRL 2638) was prepared in RPMI 1640 HG medium (Sigma-Aldrich®), treated with an antibiotic (1%), supplemented with fetal bovine serum (10%), and maintained at 37°C, in a controlled atmosphere of 5% CO_2_, as instructed in the datasheet. After reaching 80% confluence, cells were washed with phosphate buffer pH 7.4 (PBS), trypsinized, and centrifuged.

Cell plating was performed with 1.2 × 10^4^ cells/well with cell viability greater than 95% and treated with plant extracts at concentrations of 500, 1000, and 2000 *μ*g/mL. After 24 hours of treatment, the culture medium with extract was removed and washed with PBS, preserving the treated cells on the plate, where 90 *μ*L of RPMI 1640 without phenol red and 10 *μ*L of [3-(4,5-dimethylthiazol-2yl)-2,5-diphenyltetrazolium] solution (MTT solution) were added to each well and incubated for 4 hours, at 37°C, in a controlled atmosphere of 5% of CO_2_. After the incubation period, 75 *μ*L of the medium from each well was removed, and then, DMSO reagent (50 *μ*L/well) was added to solubilize the formazan crystals generated in the assay. The plate was reincubated for 10 minutes under the same conditions previously described, and then, the reading was performed on a spectrophotometer (Victor®) at a wavelength of 540 nm. The MTT method consists of a colorimetric assay to evaluate cell viability, being highly sensitive to cytotoxic agents [36]. All tests were performed in triplicate. Results were expressed as % cell viability.

### 2.8. Statistical Analysis

The results were expressed as mean and standard deviation of the mean. Analysis of variance was performed using one-way ANOVA, followed by Tukey's post hoc test, and was considered significant when *p* < 0.05, in statistical software GraphPad Prism 9.

## 3. Results and Discussion

### 3.1. Phytochemical Analysis

The assay carried out by UHPLC-QTOF MS made it possible to find 30 main peaks for the methanolic extract of *E. plana* (Table 1) and 20 for the methanolic extract of *T. catharinensis* (Table 2). From the mass (m/z) obtained from UHPLC-QTOF MS data analysis and structure through the software Data Analysis 4.2, we suggested the identification of the compound and obtained through data available in these databases (PubChem®, massbank.eu). The mass spectra obtained in the assay are presented in Figure S1 (*E. plana*) and Figure S2 (*T. catharinensis*), in supplementary materials, and follow the order presented in the respective tables.

The phytochemical compounds suggested for *E. plana* extract are vanillic acid, caffeic acid, quinic acid, quinoline, benzylmorphine, tamoxifen, and p‐coumaric-o‐glucoside, among others.

Caffeic acid is a phenolic compound of great relevance present in a wide range of plant species. Tests using the compound in isolation reveal antioxidant, anti-inflammatory, antimicrobial, and antitumor properties, with a mechanism of action of the molecular pathway of oncogenesis [37, 38]. This compound is also described in phytochemical investigation assays of extracts obtained from different parts of the same plant (*E. plana*), using other extraction methods, and employing methodological analysis such as thin-layer chromatography and high-performance liquid chromatography [32]. Caffeic acid and its derivatives are reported to have effects against several types of cancer (breast, hepatocellular carcinoma, skin, lung, oral, and cervical) [37].

Favaretto and collaborators [39], who are pioneers in studying this plant, identified new compounds of the diterpeno-cassene type: neocassa-1,12 (13), 15-triene-3,14-dione, 1,19-norneocassa-1.12 (13), 15-triene-3,14-dione, 2 and 14-hydroxyneocassa-1.12 (17), 15-trien-3-one, 3 and identified extract fractions (methanol and dichloromethane) from the roots. The compounds were able to inhibit the growth of duckweed by 50%, and these compounds in isolation showed good response against fungi that seem to be closely related to the allelopathic profile of the plant. The possibility of working with extract fractions containing these compounds in isolation significantly increased the response to the treatments.

With the exception of the previously described phenolic compounds [12, 38], our study suggests the presence of more than 20 compounds that had not yet been related to *E. plana*. The phytochemical constituent number 6 (m/z 371.2406, presented in table 1) and peak 6 in Figure S1, was identified as the compound Tamoxifen, already used as a chemotherapy drug for the treatment of breast cancer [40].

Interestingly, peak number 7 (m/z 375.1841), in Figure S1, indicates the presence of a compound identified as benzylmorphine, a morphine derivative, which, in turn, is known worldwide for having a sedative and potent analgesic effect, one of the first effective drugs to treat pain in people suffering from cancer, for example [41]. This is the first report of the presence of this molecule in *E. plana* extract.

Also, peak 1 (m/z 163.0389), which can be observed both in the *E. plana* extract and in the *T. catharinensis* extract, indicates the presence of a heterocyclic azole compound, and the class is widely used in the treatment of infections fungicides and also as a microbial and antitumor agent [42].

Among the compounds suggested for the *T catharinensis* extract, we highlight the following: compounds of the phenolic class (vanillic acid and quinic acid), where these compounds are present in a wide variety of fruits, vegetables, and herbs and are often studied due to their potential pharmacological use [43].

Alkaloids suggested for the *T catharinensis*, among which alstovenine, rhynchophylline, amezepine, voacangine, and coronaridine already have biological activity described in the literature [44]. Rhynchophylline, identified in Figure S2, in peak 8, has a cytotoxic activity for tumor cells, being able to reduce resistance to chemotherapy by a mechanism of decreased efflux of drugs via inhibition of the ATP-binding cassette (ABCB1) transporter, reducing multidrug resistance to chemotherapy [45].

Other phytochemical studies involving *Tabernaemontana* also indicate the presence of some alkaloids suggested in our study. The present voacangine and coronaridine are described in different plants of the *Tabernaemontana* genus. These alkaloids are related to the strong antimicrobial activity of the plant, in addition to in vitro antitumor and anticholinesterase activity, whether isolated or synergistically with the other constituents [46–48].

### 3.2. Antibacterial Activity

The methanolic extract had bactericidal activity against all tested microorganisms, in all tested concentrations. The extract of *E. plana* showed the lowest MIC (15.3 *μ*g/mL) found for *Staphylococcus aureus* subsp. *aureus* ATCC® 25923, an important Gram-positive pathogen that is involved in many infectious processes [49]. The extract of *Tabernaemontana catharinensis* A DC has the lowest inhibitory concentration (3.13 *μ*g/mL) as it was observed against MO *Staphylococcus aureus* subsp. *aureus* ATCC® 29213, and a Gram-positive, methicillin-resistant pathogenic microorganism is associated with a wide number of infections, including necrotizing pneumonia [50]. The results for both extracts are shown in Table 3.

Other study tested the methanolic extract of roots and aerial parts of grass annoni against *Staphylococcus aureus* (ATCC 27213), *Staphylococcus epidermidis* (ATCC 12228), *Pseudomonas aeruginosa* (ATCC 27853), and *Salmonella typhi* (ATCC 19196), finding an MIC of 100 mg/mL only for MO treated with the extract prepared with the aerial part of the plant, a concentration much higher than that presented in our study. The authors report that there was no inhibition of bacterial growth compared to extracts from the plant's roots, suggesting that most of the metabolites are present in the aerial parts of the plant [13].

Gindri et al. [51] evaluated the activity of crude ethanolic extract and three fractions of *T. catharinensis* against the following MO: *Micrococcus* sp., *Staphylococcus aureus* and *Pseudomonas aeruginosa*, *Escherichia coli*, *Klebsiella pneumoniae*, *Aeromonas* sp. (all with MIC >1000 mg/mL), *Enterococcus faecalis*, and *Proteus mirabilis* (MIC: 62.50 mg/mL), where only the n-butanol fraction showed some antimicrobial activity. The authors suggest that this result is related to polar compounds (phenolic compounds, tannins, and flavonoids) present in the extract fraction and that these, in turn, have greater microbiological activity. Our results, using crude extract, present antimicrobial activity at lower concentrations than that indicated in the cited study, being less than 500 *µ*g/mL for both extracts, emphasizing the potential use of plants as an antimicrobial agent. The solvent chosen for preparing and obtaining the crude extract seems to be closely related to the increase or decrease in antimicrobial activity [52, 53].

The antibacterial activity presented by the *E. plana* and *T. catharinensis* extracts may be related to the presence of phenolic compounds such as caffeic acid [37], vanillic acid [54], quinic acid [55], p-coumaric acid [56, 57], and, also the presence of alkaloids such as voacangine [58], coronaridine [59], and quinoline [60], identified through phytochemical analysis. Studies involving these isolated compounds are of future interest, aiming to better elucidate their effects.

### 3.3. *In Vitro* Hemolysis Assay

**Figure 1:**
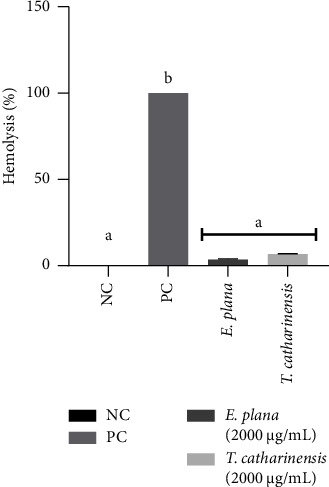
Percentage of hemolysis of erythrocytes exposed to treatment with *Eragrostis plana* NEES and *Tabernaemontana catharinensis* A DC extracts, at a concentration of 2000 *µ*g/mL. NC: negative control; PC: positive control. Results were expressed as mean and standard deviation. Different letters mean statistically significant difference (*p* < 0.05). Assays were performed in triplicate.

Both extracts tested did not cause hemolysis in erythrocytes (Figure 1) when tested at a concentration of 2000 µg/mL, and the concentration chosen for the test contemplates the concentrations used in the other tests of the present study. The hemolysis assay is used to assess the toxicity of compounds *in vitro*, in a stage prior to *in vivo* assays [34]. The range of protocols to assess hemolysis is a bias that hinders the comparison between results. A recent study proposes a standardization for the *in vitro* hemolysis assay to assess cytotoxicity, and this standardized protocol will facilitate the comparison between studies [61].

### 3.4. Cell Culture and Viability

**Figure 2:**
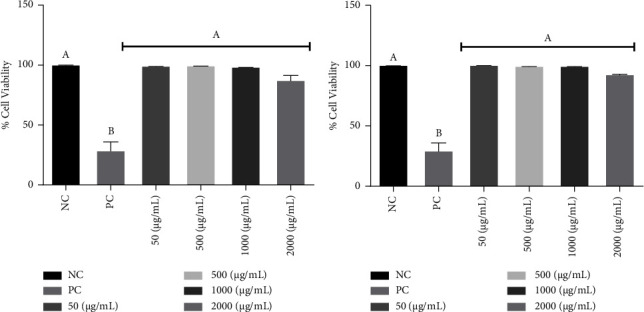
Percentage of viable PBMCs exposed to treatment with *Eragrostis plana* NEES (a) and *Tabernaemontana catharinensis* A DC (b) extract at different concentrations. NC: negative control; PC: positive control (H_2_O_2_). Results were expressed as mean and standard deviation. Different letters mean a statistically significant difference (*p* < 0.05). 03 assays in triplicate were performed.

The extracts showed no cytotoxic effect on the PBMC culture *in vitro*, at any of the concentrations tested (Figures 2(a) and 2(b)), and this is one of the main objectives when looking for a new therapeutic agent, which is efficient without affecting healthy cells, enabling the expansion of the range of the assay.

**Figure 3:**
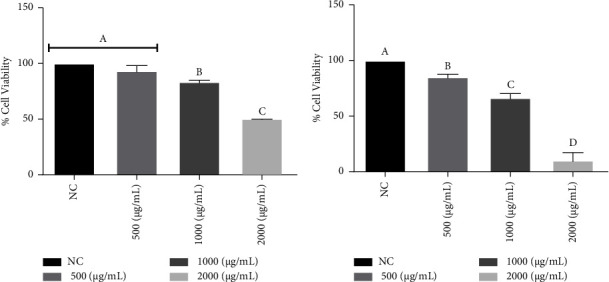
Percentage of viable cells (CT26) *in vitro* exposed to treatment with methanolic extract of *Eragrostis plana* NEES (a) and *Tabernaemontana catharinensis* A DC (b) at different concentrations. NC: negative control; results were expressed as mean and standard deviation. Different letters mean a statistically significant difference (*p* < 0.05). 03 assays in triplicate were performed.

**Figure 4:**
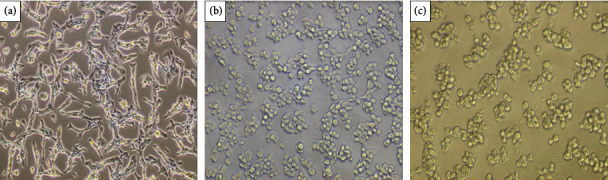
Representative image of morphological changes in CT26 cells treated with *Eragrostis plan*a NEES extract. (a) Untreated cells (NC); (b) cells treated at the concentration of 1000 *μ*g/mL; (c) cells treated at the concentration of 2000 *μ*g/mL.

**Figure 5:**
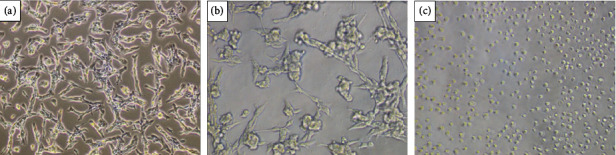
Representative image of morphological changes in CT26 cells treated with *Tabernaemontana catharinensis* A DC extract. (a) Untreated cells (NC); (b) cells treated at the concentration of 1000 *μ*g/mL; (c) cells treated at the concentration of 2000 *μ*g/mL.

On the other hand, both extracts decreased the cell viability of the CT26 cell line *in vitro* at all concentrations tested, as shown in Figures 3(a) and 3(b). Furthermore, a morphological change in the cells (Figure 4 and 5) was observed at concentrations of 1000 (b) and 2000 (c) *μ*g/mL. The negative control group (a), untreated cells, maintained high viability and did not have its morphology altered, which allows us to state that the result is in fact related to the treatment with the extracts, being this the first report of *in vitro* assays of the extract *E. plana* in front of a tumor lineage.

Artico et al. [62] evaluated the cytotoxic, genotoxic, and mutagenic potential of two extracts of *E. plana* in the experimental model of *Allium cepa*. The extract used as a positive control (PC) was obtained from *E. plana* collected in a region of charcoal-contaminated soil, and the other (negative control) obtained from *E. plana* was collected in a region with a low probability of contamination. The *E. plana* extract obtained from plants in contaminated (PC) soil showed a greater mutagenic and genotoxic potential, although both had an antiproliferative effect. The authors attribute the fact that the extract had this effect due to the large number of secondary metabolites produced by the plant, as evidenced by Favaretto and collaborators in their study on the plant's phytochemistry [32].

The toxic effect of fractions of the extract obtained from the bark and roots of *T. catharinensis* was tested against tumor cell lines (HeLa; B-16; Hep-2) and a nontumor cell line of 3T3 fibroblasts. The authors found a cytotoxic effect against tumor cell lines; however, the same result was identified in normal cells [23]. Rosales et al. [22] identified the cytotoxic activity of an alkaloid isolated from the ethanol extract of *T. catharinensis* bark against human melanoma cell lines (A375, WM1366, and SK-MEL-28). Similarly, others report the antiproliferative effect of the alkaloid fraction also isolated from the bark of the plant, against a human colorectal cancer strain (HT-29), with a low toxic effect in nontumor strains [24].

The antitumor effects found by the action of the *E. plana* and *T. catharinensis* extracts may be related to the compounds, suggested by the phytochemical analysis. For *E. plana* extracts, we suggest caffeic acid [37, 38], benzothiazole derivatives [42], and tamoxifen [40] which is already used in clinical medicine as chemotherapy. Quinoline alkaloids and derivatives have anticancer activity [60, 63]. For *T. catharinensis* extracts, the presence of some alkaloids reinforces these actions, such as voacangine, coronaridine [47, 48], and rhynchophylla, and exhibits cytotoxicity towards tumor cell lines [45]. Vanillic acid has activity against colon cancer cells (HCT 116) [64] and quinic acid in nanoparticles against solid tumors [65, 66].

Considering that we used the crude extract of *T. catharinensis*, our results corroborate the findings in the literature, evidencing the antitumor potential of the genus *Tabernaemontana.* Nevertheless, it was possible to observe that the extract caused morphological changes in the CT26 strain. We suggest that these changes were related to the decrease in cell viability.

Furthermore*, Tabernaemontana catharinensis* extract had the lowest inhibitory concentration (3.13 *μ*g/mL) against MO *Staphylococcus aureus* subsp. *aureus* ATCC® 29213, a Gram-positive, methicillin-resistant pathogenic microorganism, compared to *E. plana* extract (245 *μ*g/mL), and for *Enterococcus faecalis* ATCC® 29212, the MIC was 3.13 *μ*g/mL while for *E. plana*, a MIC of 30.6 *μ*g/mL. Against most MOs tested, *T*. *catharinensis* extract had a lower MIC than *E. plana* extract. In addition, *Tabernaemontana catharinensis* extract shows a higher cytotoxicity in CT26 cells, from 500 *μ*g/mL, whereas *E. plana* extract was from 1000 *μ*g/mL, showing its superior antineoplastic effect.

## 4. Conclusion

The methanolic extracts of *E. plana* and *T. catharines* showed antiproliferative effect against tumor lineage (CT26), as well as antibacterial effect against different pathogenic strains (MIC <500 *µ*g/mL), without showing cytotoxic effect in PBMCs and hemolysis in erythrocytes. The antibacterial and antineoplastic activities of the extracts showed a cytosafe concentration. The phytochemical constitution suggested for both extracts, with compounds already known to exert antitumor and antibacterial activity, and possibly the synergism between them, can be involved in the results shown in this study. Our study is the first to show that the crude extract of *E. plana* is effective against human pathogens, as well as the CT26 tumor lineage, without causing toxic effects in the immune system cells. Studies should be carried out to verify possible molecular targets involved in the antitumor effect *in vivo*.

## Figures and Tables

**Table 1:** Mass/charge (m/z), suggested structural formula of possible phytochemical constituents in *E. plana* extract obtained through UHPLC-QTOF MS analysis.

*Eragrostis plana* NEES extract				
Peak	Meas (m/z)	Íon formula	(m/z)	Likely compound
1	163.0389	C_7_H_5_N_3_O_2_	163.0389	1H-Benzotriazole-5-carboxylic acid
2	172.0944	C_7_H_12_O_4_	172.0944	Glycylproline
3	181.0496	C_9_H_8_O_4_	181.0495	Caffeic acid
4	181.0507	C_9_H_9_O_4_	181.0506	Vanillic acid
5	191.0558	C_7_H_11_NO_6_	191.0558	Quinic acid
6	371.2406	C_26_H_29_NO	371.2406	Tamoxifen
7	375.1841	C_24_H_25_NO_3_	375.184	Benzyl morphine
8	377.1963	—	377.1963	Unidentified
9	385.1622	C_17_H_27_N_3_O_5_S_5_	385.1622	Amisulpride N-oxide
10	129.0524	C_9_H_7_N	129.0524	Quinoline
11	267.0539	C_12_H_13_NO_4_S	267.0539	Oxycarboxyn
12	356.2795	C_18_H_36_N_4_O_3_	356.2782	Unidentified
13	399.1779	C_22_H_29_N_3_S_2_	399.1779	Thiethylperazine
14	537.3142	C_21_H_36_N_15_NaO	537.3119	Unidentified
15	479.2616	C_22_H_35_N_6_O_6_	479.2613	Unidentified
16	141.9585	C_2_H_6_O_3_S_2_	141.9585	2-Mercaptoethanesulfonic acid
17	241.2034	C_12_H_25_N_4_O	241.2023	4-Acetamidobutyl-(diaminomethylidene)-(3-methylbut-2-eny)azanium
18	342.2641	C_20_H_32_N_5_	342.2652	1-N,1-N′-bis[2-(dimethylamino) ethyl]-2-(1-methylquinolin-1-io-2-il) eteno-1,1-diamina
19	325.2275	C_21_H_29_N_2_O	325.2274	p-Coumaric acid 4-o-glucoside
20	356.2804	C_20_H_38_NO_4_	356.2795	2-(Diocthylamino)-2-oxoetoxiacetato
21	246.2427	C_14_H_32_NO_2_	246.2428	Bis(6-hydroxyhexyl)-dimethylazanium
22	370.2953	C_21_H_40_NO_4_	370.2952	2-Butenedioic acid 1-dodecyl 4-[2-(trimethylamino) ethyl] ester
23	323.1599	C_14_H_21_N_5_O_4_	323.1599	O (6)-*n*-Butyldexyguanosine
24	341.1088	C_12_H_21_O_11_	341.1089	3,4-Di-hydroxi-6-(hydroxymethyl)-5-[3,4,5-tri-hidroxi-6-(hydroximetil) oxan-2-il] oxioxan-2-olato
25	387.1143	C_13_H_23_O_13_	387.1144	2,3,4,5,6-Pentahydroxy7 [(2S,3R,4S,5S,6R)-3,4,5-trihydroxy-6-(hydroxymethyl) oxan-2-yl] oxyheptanoic acid
26	371.0973	C_14_H_7_N_14_	371.0984	Unidentified
27	579.1344	C_26_H_27_O_15_	579.1355	Cyanidin-3-xyloglucoside
28	563.1393	C_25_H_21_N_7_O_9_	536.1406	Ethyl2-[6-(5-cyano-2-nitrophenoxy)-2-[3-(1-methyl-4,5-dihydroimidazol-2-yl) phenoxy]-5-nitropyrimidin-4-yl] oxyacetate
29	401.1807	C_18_H_23_N_7_O_4_	401.1817	4-[[(4,6-Dimorfolin-4-il-1,3,5-triazin-2-il) hydrazinilideno] methyl] benzene-1,2-diol
30	689.4067	C_28_H_59_N_5_O_14_	689.4063	(2S,3S,4S)-5-Amino-N-[(2R)-5-[(2R,6S)-3-amino-6-[(2,3,4-trihydroxybutylamino) methyl] oxan-2-yl] oxy-3-[(1R,3S)-2,4-dihydroxy-1-methoxy-3-(methylamino) pentoxy]-4-hydroxyhexan-2-yl]-2,3,4-trihydroxypentanamide

**Table 2:** Mass/charge (m/z), suggested structural formula of possible phytochemical constituents in *T. catharinensis* extract obtained through UHPLC-QTOF MS analysis.

*Tabernaemontana catharinensis* A DC extract				
Peak	Meas (m/z)	Ion formula	(m/z)	Likely compound
1	163.0389	C_7_H_5_N_3_O_2_	163.0389	1H-Benzotriazole-5-carboxylic acid
2	181.0507	C_9_H_9_O_4_	181.0506	Vanillic acid
3	191.0558	C_7_H_11_O_6_	191.0561	Quinic acid
4	375.1841	C_24_H2_5_NO_3_	375.184	Benzylmorphine
5	377.1952	C_20_H_28_N_2_O_5_	377.1952	1-(2-{[1-(Ethoxycarbonyl)-3-phenylpropyl]amino}propanoyl) proline maleate
6	378.1987	C_21_H_30_O_6_	378.1987	Unidentified
7	353.1848	C_21_H_25_N_2_O_3_	353.186	Alstovenine
8	385.2119	C_22_H_29_N_2_O_4_	385.2122	Rhynchophylline
9	265.1698	C1_8_H_21_N_2_	265.1699	Amezepine
10	255.2191	C_13_H_27_N_4_O	255.2179	(5R)-4-[(2R)-Butan-2-yl]-5-methyl-6-oxo-5H-1,2,4-triazin-1-yl] methyl-diethylazanium
11	356.2795	C_18_H_36_N_4_O_3_	356.2782	2-[Bis [2-(butylamino)-2-oxoethyl] amino]-N-butylacetamide
12	537.3142	C_21_H_36_N_15_NaO	537.3119	Unidentified
13	479.2616	C_22_H_35_N_6_O_6_	479.2613	Unidentified
14	369.2171	C_22_H_28_N_2_O_3_	369.2173	Voacangine
15	355.2009	C_21_H_27_N_2_O_3_	355.2016	Coronaridine hydroxyindolenine
16	339.2063	C_21_H_26_N_2_O_2_	339.2067	Coronaridine
17	559.3229	C_24_H_49_NO_13_	559.3209	AminoPeg-11
18	769.2175	C_35_H_37_N_4_O_16_	769.221	Isorhamnetin 3-orhamnoside-7-O-(rhamnosyl-hexoside)
19	401.1449	C_16_H_23_N_3_O_9_	401.144	2-Amino-2-[5-(3-carboxypropyl)-1-methyl-2,4,6-trioxo-1,3-diazinan-5-yl] heptanedioic acid
20	451.2182	C_20_H_35_O_11_	451.2185	2-[[5,7-Dihydroxy-2-(3,4,5-trihydroxycyclohexyl)-3,4,4a,5,6,7,8,8a-octahydro-2H-chromen-1-ium-3-yl] oxy] oxane-3,4,5-triol

**Table 3:** Minimum inhibitory concentration (MIC) of the extract of *Eragrostis plana* NEES and *Tabernaemontana catharinensis* A DC, against different microorganisms.

Microorganism	*E. plana* MIC (*μ*g/mL)	*T. catharinensis* MIC (*μ*g/mL)
*Acinetobacter baumannii Bouvet and Grimont* ATCC ® BAA-747	30.6	25
*Pseudomonas aeruginosa (Schroeter) Migula* ATCC ® 27853	30.6	12.5
*Shigella* spp (clinically isolated)	122.5	50
*Klebsiella pneumoniae* subsp. *pneumoniae* ATCC® 700603	122.5	50
*Escherichia coli* ATCC® 25922	122.5	100
*Proteus mirabilis* (clinically isolated)	490	50
*Salmonella* spp (clinically isolated)	490	401
*Staphylococcus aureus* subsp. *aureus* ATCC® 25923	15.3	25
*Staphylococcus saprophyticus* ATCC® bAA-750	61.25	200
*Staphylococcus aureus* subsp. *aureus* ATCC® 29213	245	3.13
*Enterococcus faecalis* ATCC® 29212	30.6	3.13
*Enterococcus casseliflavus* ATCC® 700327	122.5	200
*Streptococcus pyogenes* (clinically isolated)	122.5	200

## Data Availability

The data used to support the findings of this study are available from the corresponding author upon request (e-mail: clecim2@gmail.com).

## Supplementary Materials

Supplementary Materials Figure S1: Mass spectrum for the extract of *Eragrostis plana* NEES, obtained through UHPLC-QTOF MS analysis. Figure S2: Mass spectrum for the extract of *Tabernaemontana catharinensis* A DC, obtained through UHPLC-QTOF MS analysis.
