# Supplementary material for: Phytochemistry Profile, Antimicrobial and Antitumor Potential of the Methanolic Extract of Tabernaemontana catharinensis A DC and Eragrostis plana NEES
Source: Evid Based Complement Alternat Med. 2024 Jan 3;2024:5513141. doi: 10.1155/2024/5513141 (PMC10781527; doi:10.1155/2024/5513141)
Supplement: Supplementary Materials — Figure S1: Mass spectrum for the extract of Eragrostis plana NEES, obtained through UHPLC-QTOF MS analysis. Figure S2: Mass spectrum for the extract of Tabernaemontana catharinensis A DC, obtained through UHPLC-QTOF MS analysis. [file 5513141.f1.zip › Supplementary file. Figure S2. Phytochemistry Profile.docx]

**Phytochemistry Profile, Antimicrobial and Antitumor Potential of the Methanolic Extract of *Tabernaemonthana catharinensis* A DC and *Eragrostis plana* NEES**

Emanoeli da Rosa,^1^ Cheila Denise Ottonelli Stopiglia,^2^ Michel Mansur Machado,^2^ Augusto Cezar Dotta Filho,^1^ Ursula Paula Reno Soci,^3^ Andreas Sebastian Loureiro Mendez,^4^ Tiago Fernandes,^3^ Edilamar Menezes de Oliveira,^3^ Cleci Menezes Moreira.^1^

1. Programa de Pós-Graduação em Bioquímica, Universidade Federal do Pampa - UNIPAMPA, Uruguaiana, RS, Brazil;

2. Programa de Pós-Graduação em Ciências Farmacêuticas, Universidade Federal do Pampa- UNIPAMPA, Uruguaiana, RS, Brazil;

3. Laboratório de Bioquímica e Biologia Molecular do Exercício, Escola de Educação Física e Esporte - Universidade de São Paulo - USP, SP, Brazil.

4. Programa de Pós-Graduação em Ciências Farmacêuticas, Universidade Federal do Rio Grande do Sul - UFRGS, Porto Alegre, RS, Brazil.

**
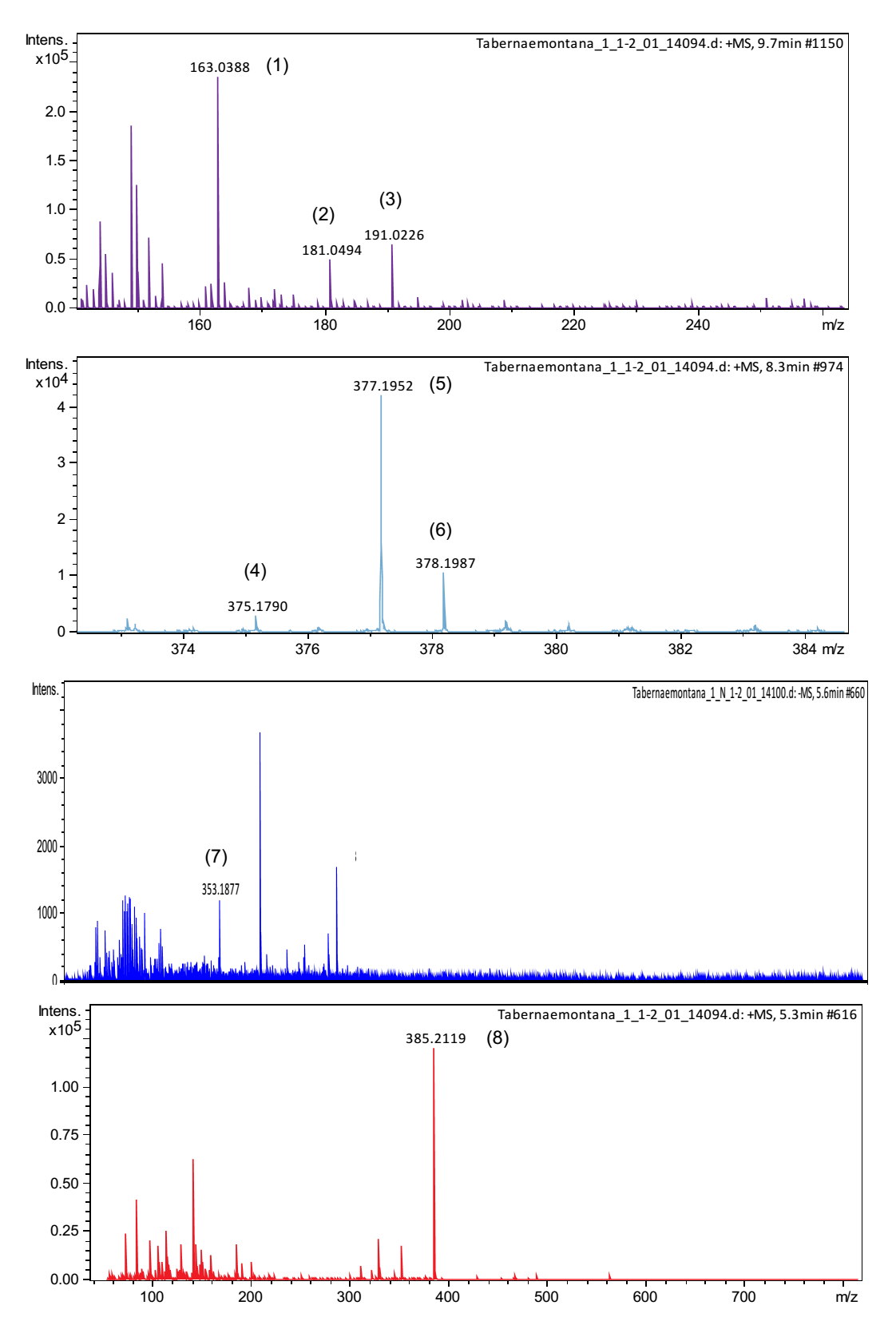
**

**
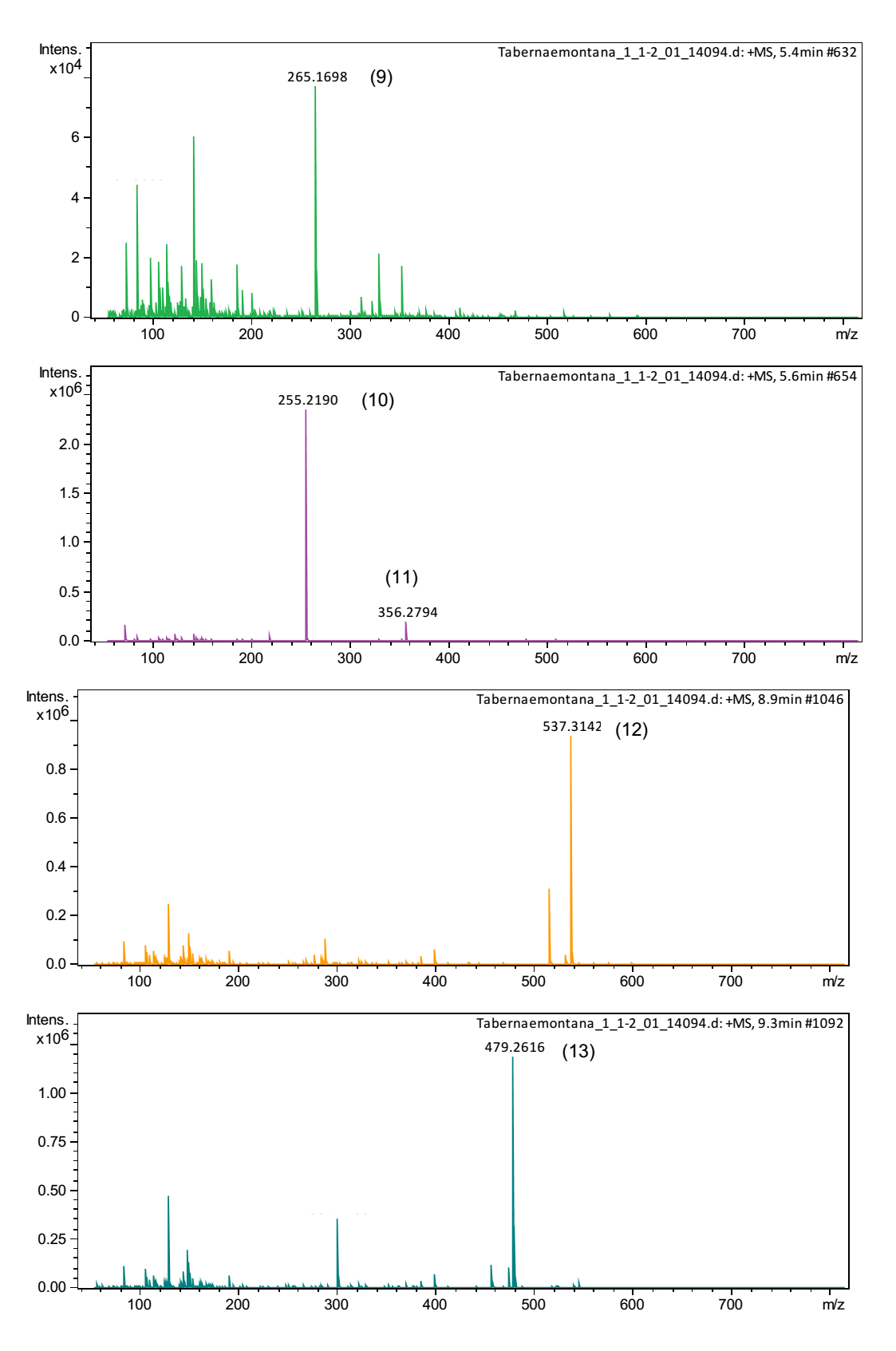
**

**
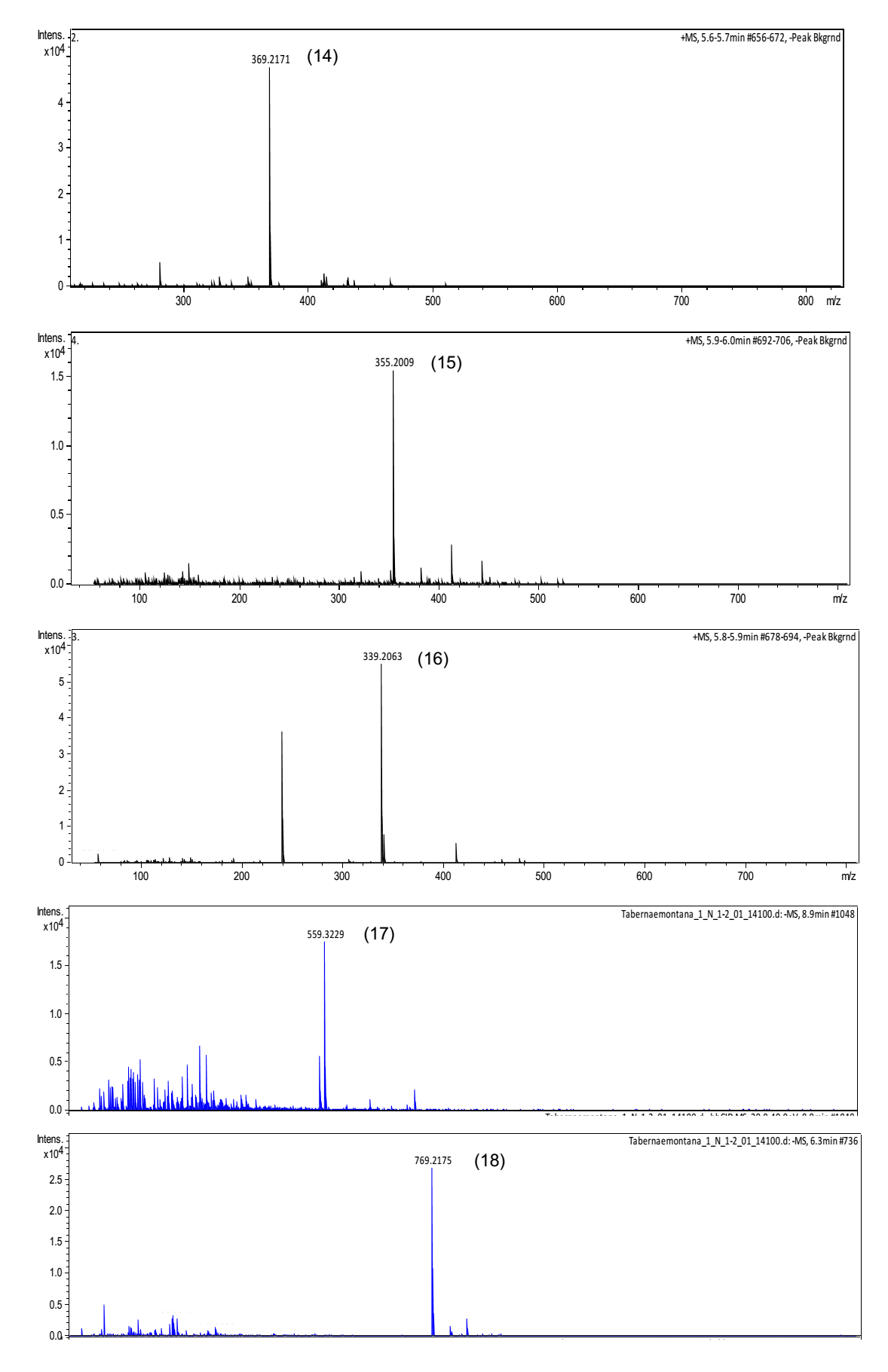
**

**
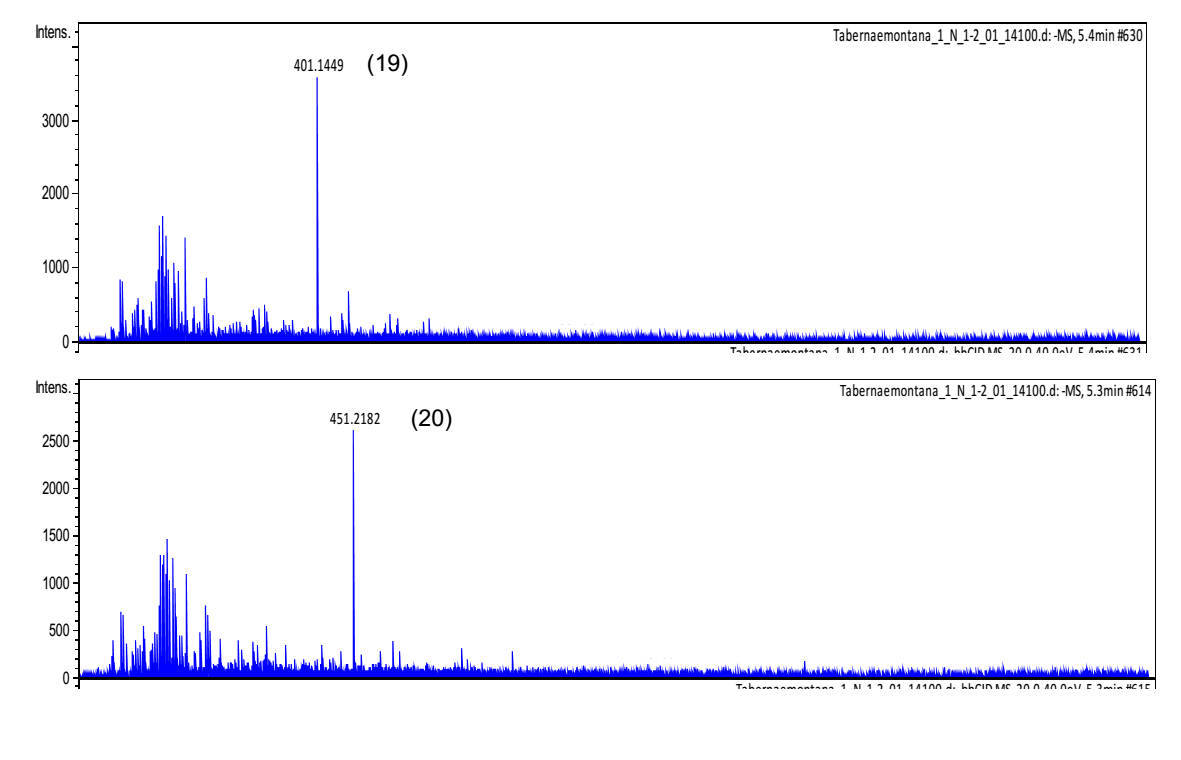
**

**Figure S2**: Mass spectrum for the extract *Tabernaemontana catharinensis* A DC, obtained through UHPLC-QTOF MS analysis. The numbers in parentheses correspond to the numbering of compounds in table 2.
